# Identification of drought tolerant mechanisms in a drought-tolerant maize mutant based on physiological, biochemical and transcriptomic analyses

**DOI:** 10.1186/s12870-020-02526-w

**Published:** 2020-07-09

**Authors:** Qinbin Zhang, Hui Liu, Xiaolin Wu, Wei Wang

**Affiliations:** College of Life Sciences, National Key Laboratory of Wheat and Maize Crop Science, Henan Agricultural University, Zhengzhou, China

**Keywords:** Differentially expressed genes, Drought-adaptation model, Drought-tolerant mutant, Drought stress, Transcriptomic analysis, *Zea mays*

## Abstract

**Background:**

Frequently occurring drought stress negatively affects the production of maize worldwide. Numerous efforts have been made to develop drought-tolerant maize lines and to explore drought tolerant mechanisms in maize. However, there is a lack of comparative studies on transcriptomic changes between drought-tolerant and control maize lines.

**Results:**

In the present study, we have developed a drought-tolerant maize mutant (C7–2t) by irradiating the seeds of maize inbred line ChangC7–2 (C7–2) with ^60^Co-γ. Compared to its wild type C7–2, C7–2t exhibited a significantly delayed wilting and higher drought tolerance under both the controlled and field conditions, indicating its high water-holding ability. Transcriptomic profiling was performed to identify differentially expressed genes (DEGs) between C7–2 and C7–2t during drought. As a result, a total of 4552 DEGs were implied in drought tolerance of C7-2 and C7-2t. In particular, the expression of photosynthesis-related genes in C7–2 was inhibited, whereas these genes in C7–2t were almost unaffected under drought. Moreover, a specific set of the DEGs were involved in phenylpropanoid biosynthesis and taurine (hypotaurine) metabolism in C7–2t; these DEGs were enriched in cell components associated with membrane systems and cell wall biosynthesis.

**Conclusions:**

The drought tolerance of C7–2t was largely due to its high water-holding ability, stable photosynthesis (for supporting osmoregulation) and strengthened biosynthesis of cell walls under drought conditions.

## Background

Frequently occurring drought stress negatively affects the production of maize worldwide [1, 2]. Enhancing maize resistance to drought is an effective way to address the problem of yield loss caused by drought stress [3, 4]. Breeders and agronomists are interested in developing drought-tolerant lines and uncovering their drought-tolerant characteristics [5]. Therefore, it is of considerable significance to clarify the mechanisms of maize response to drought stress and adaptation to meet the increasing food demands [6].

The drought tolerance in plants is usually evaluated according to differences in phenotypes and physiological and biochemical responses [7, 8]. Drought-tolerant plants can maintain stable morphological structures during prolonged periods of drought stress by high water-holding ability [9, 10]. On the one hand, drought-tolerant lines need to enhance the ability of their roots to absorb water from soil [9]. By reducing the number and increasing the volume of individual cortical cells in maize, the root system can reduce its exploration of the surface soil and grow into deeper soil, which allows maize to obtain more water from soil [11, 12]. On the other hand, drought tolerance can be enhanced by reducing the density and controlling the opening of stomata in leaves [13, 14]. Moreover, plants can synthesize proline and soluble sugars to reduce the water potential in cells and maintain cell homeostasis by osmoregulation under drought conditions [15, 16]. Drought stress also activates the peroxide-scavenging enzyme system to remove excess reactive oxygen species (ROS) induced by drought, which can damage the cell membrane systems and eventually cause cell death [17, 18]. The activity of the antioxidant (enzymatic and nonenzymatic) system represents an effective index to evaluate drought tolerance in maize [19, 20]. Drought-tolerant maize lines have been identified by comparing physiological and biochemical changes among hybrids, inbred lines and transgenic lines [21–23]. However, these changes could not fully explain the drought tolerance in maize due to variations in genetic backgrounds. It has been well documented that drought response of plants involves a complex regulatory network; therefore, omic studies are particularly required for functional characterization of key drought-response genes to improve drought-tolerant traits in crop plants [24].

To date only a few drought-tolerant genes such as *ZmVPP1* and *ZmPP2C* in maize were identified by traditional sequencing methods and functionally characterized [22, 25, 26]. Many key genes were implied in drought tolerance in maize [22, 26–29]; however, a relative long period was required to clarify the functions of these drought-response genes [25].

With the advantages of low cost, high throughput and high sensitivity, RNA-seq is a powerful tool for the large-scale identification of drought-responsive genes and can facilitate the mining of key drought tolerance genes in plants, e.g., maize [30–37]. For example, RNA-seq studies have shown that the upregulation of cell wall biosynthesis/aquaporin-related genes allows maize recombinant inbred lines to gain drought adaptability under drought conditions [27]; the genes related to cell wall remodeling are involved in drought-response processes in a drought-tolerant maize line, and the syntheses of amino acids and carbohydrates are related to drought tolerance [36].

In the present study, we created a drought-tolerant line (C7–2t) by irradiating seeds of maize inbred line Chang7–2 (C7–2). To explore the mechanisms of drought resistance of C7–2t, we have compared physiological, biochemical and transcriptomic changes between C7–2 and C7–2t. Our results would highlight the drought tolerance signatures of C7–2t and contribute to the identification and functional studies of novel drought tolerance genes in maize.

## Results

### Creation and screening of drought-tolerant maize mutants

**Fig. 1:**
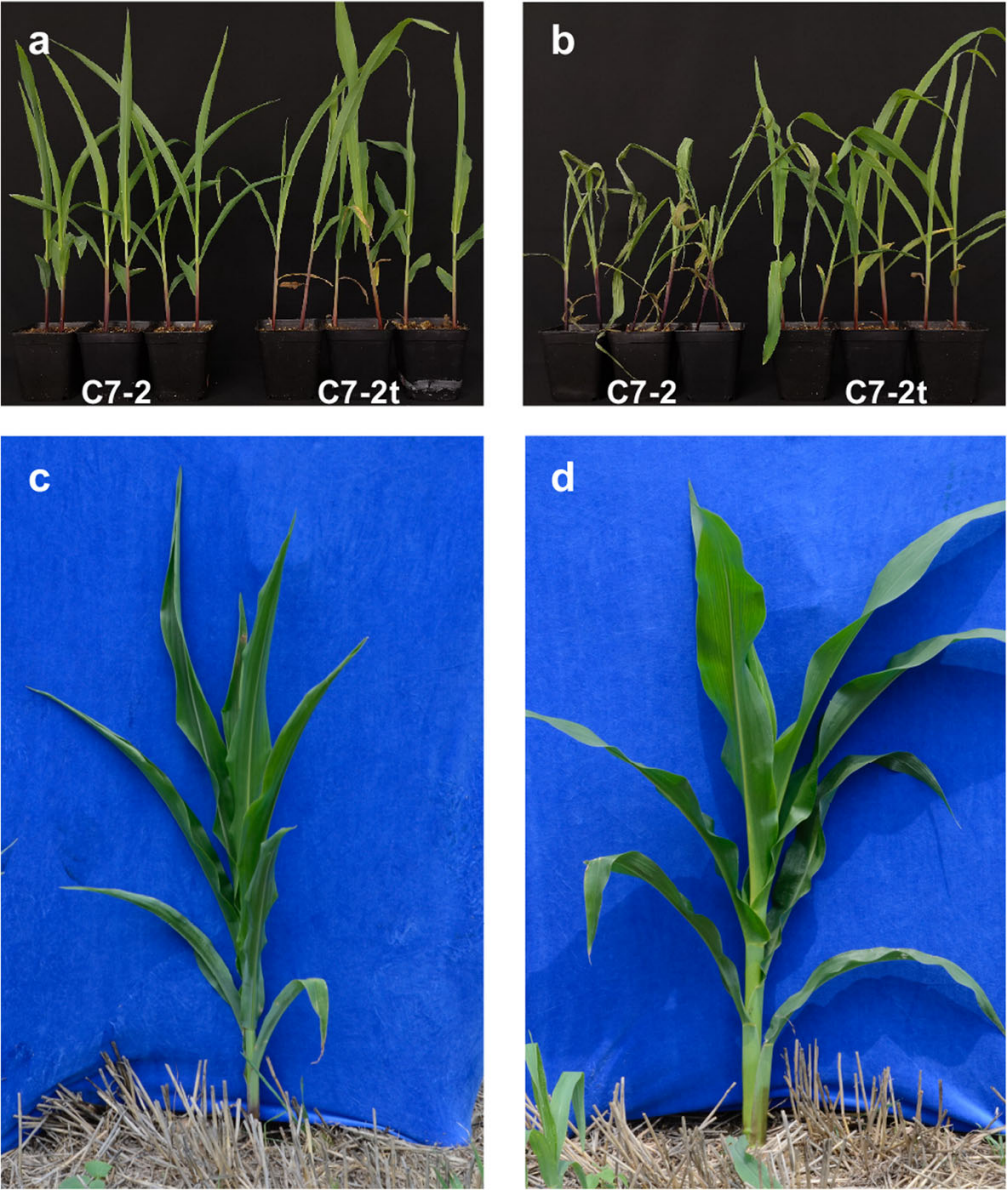
Evaluation of drought tolerance of C7–2 and C7–2t. **a** Normal watering conditions (control), 16-day-old seedlings. **b** Water withheld for 8 days (drought), 16-day-old seedlings. **c** and **d**, seedlings in the field at 35 days after sowing

The ^60^Co-γ radiated maize seeds at 200 Gy for 1 h were used for screening of drought-tolerant lines because the fatality rate of mutagenesis was 50%, i.e. about half of the treated seeds lost their germination abilities. Then, the promising drought-tolerant mutants were obtained by continuous self-crossing for five years. The laboratory study further verified that one of the mutants (C7–2t) showed an excellent drought-tolerant performance at the early seedling stage (Fig. 1a; Fig. 1b). The drought-tolerance index of the mutant C7–2t was significantly higher compared to its wild-type C7–2 (Fig. S1a). Clearly, the leaves of the 35-day-old C7–2 plants curled earlier than those of C7–2t plants, which maintained the normal leaf morphology under water defect conditions in the field (Fig. 1c; Fig. 1d). Moreover, the anthesis-silk interval (ASI) of C7–2t was significantly shorter than that of C7–2 (Fig. S1b; Fig. S1c). In addition, there was no significant difference in plant height, ear height and biomass in the fields between both lines (Fig. S2). Therefore, drought-tolerant C7–2t showed an improved performance in the field. Next, physiological, biochemical and transcriptomic analyses were performed to explore the mechanisms underlying the drought tolerance in C7–2t.

### Differential drought responses between both maize lines

**Fig. 2:**
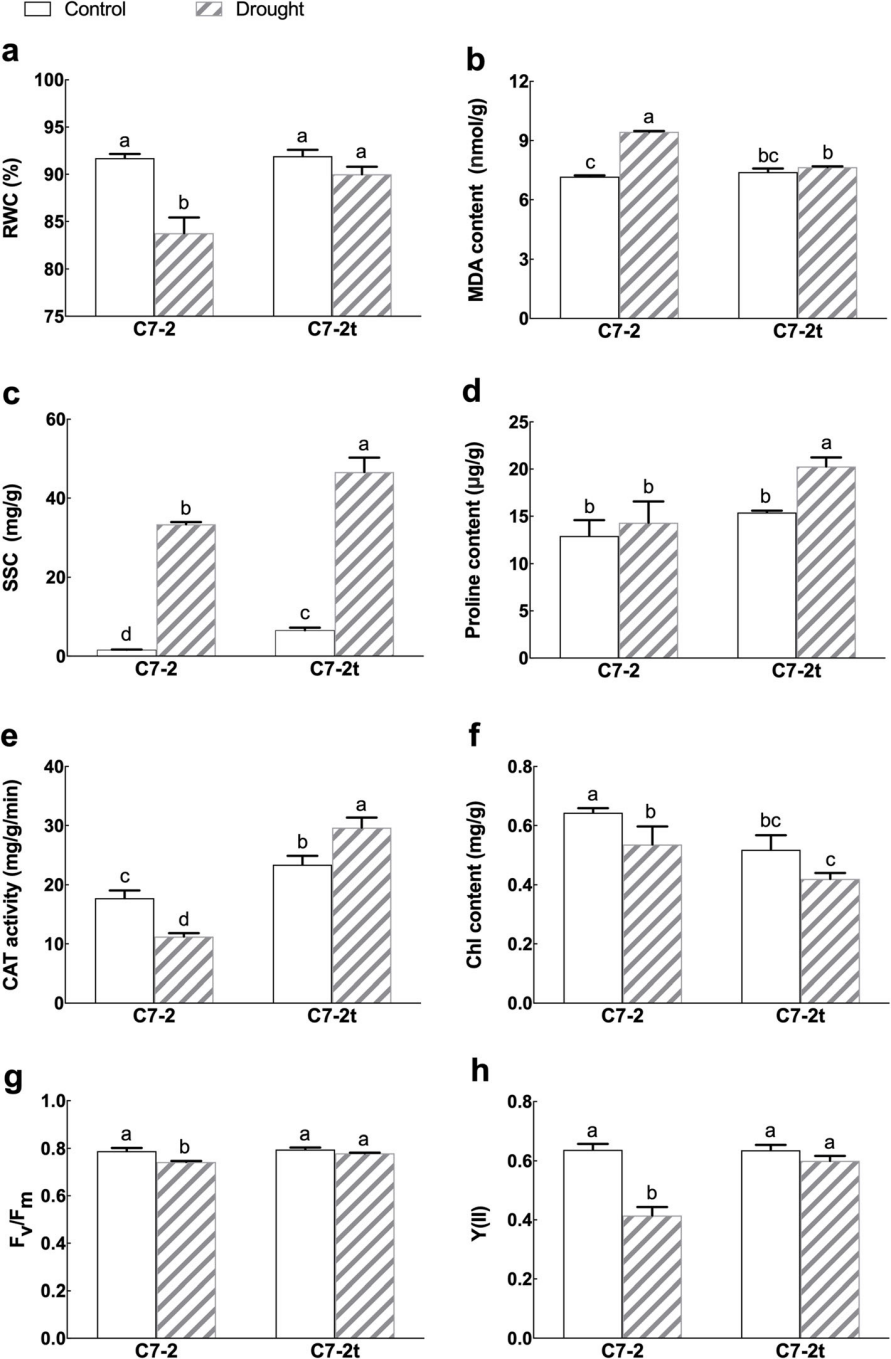
Comparison of the physiological indexes of C7–2 and C7–2t under the control and drought conditions. Different letters indicate significant difference at 0.05 significance level

Under the normal conditions, there was no significant difference in relative water content (RWC), proline content, malondialdehyde (MDA) content, or chlorophyll (Chl) fluorescence parameters between C7–2t and C7–2, whereas the catalase (CAT) activity and soluble sugar content (SSC), in the leaves of C7–2t was higher than that of C7–2 (Fig. 2).

During a 7-day drought treatment, C7–2 showed an increasingly severe wilting, whereas C7–2t did not show any signs of wilting (Fig. 1b); RWC of both lines decreased, with a more significant reduction in C7–2 than C7–2t (Fig. 2a), and the MDA content in leaves under drought was lower in C7–2t than C7–2 (Fig. 2b), suggesting C7–2t suffered less damage that C7–2 under drought stress. Moreover, the contents of SSC and proline, and the activity of CAT in C7–2t were significantly higher than those in C7–2 (Fig. 2c, d, e), implying that C7–2t was likely to enhance drought tolerance by modulation the contents of SSC and proline, and the activity of CAT.

Under drought total Chl content in the leaves of both lines decreased. It needs to note that Chl content in C7–2t was also lower than that of C7–2 under normal conditions (Fig. 2f). Clearly, the mutation has affected the Chl synthesis in the C7–2t. However, the maximum photochemical quantum yield (F_v_/F_m_) and effective photochemical quantum yield (Y(II)) of photosystem II (PS II) in C7–2t were all significantly higher than those in C7–2 under drought stress (Fig. 2g, h).

### Gene sequence assembly, annotation and categorization

For a comparison of the transcript profiles of C7–2 and C7–2t under drought stress, 12 samples from three independent biological repeats were collected for RNA-seq analysis. Raw reads of 142,060,004, 140,113,794, 134,927,492 and 141,943,752 were generated from plants in C7–2 control (CC), C7–2 drought treatment (CD), C7–2t control (TC) and C7–2t drought treatment (TD), respectively. After removal of the adapters, low-quality sequences and ambiguous reads, 138,139,372 (CC), 135,923,246 (CD), 131,095,380 (TC), and 138,366,112 (TD) clean paired-end reads were obtained, respectively (Table 1). The expression levels of the genes were evaluated using FPKM (fragments per kilobase of exon model per million reads mapped) values. The percentage of genes with FPKM ≥1 in the 12 samples ranged from 56.19 to 59.91%. There were fewer genes with FPKM > 1 in C7–2t than in C7–2. The percentage of genes with FPKM ≥100 in the 12 samples ranged from 2.09 to 2.38% (Table S1).

**Table 1:** Overview of the RNA sequencing results

Sample	CC 1	CC 2	CC 3	CD 1	CD 2	CD 3	TC 1	TC 2	TC 3	TD 1	TD 2	TD 3
Raw Reads Number	49,088,148	47,002,534	45,969,322	46,547,122	47,791,948	45,774,724	47,070,676	45,172,694	42,684,122	47,715,762	46,324,412	47,903,578
Clean Reads Number	47,579,140	45,624,176	44,936,056	45,324,662	46,358,612	44,239,972	45,444,044	44,010,808	41,640,528	46,491,316	45,179,856	46,694,940
Clean Reads Rate (%)	96.93	97.07	97.75	97.37	97	96.65	96.54	97.43	97.56	97.43	97.53	97.48
Raw Bases Number	7,363,222,200	7,050,380,100	6,895,398,300	6,982,068,300	7,168,792,200	6,866,208,600	7,060,601,400	6,775,904,100	6,402,618,300	7,157,364,300	6,948,661,800	7,185,536,700
Clean Bases Number	7,136,871,000	6,843,626,400	6,740,408,400	6,798,699,300	6,953,791,800	6,635,995,800	6,816,606,600	6,601,621,200	6,246,079,200	6,973,697,400	6,776,978,400	7,004,241,000
Mapped Reads	40,747,260	41,595,903	39,823,188	43,134,590	41,366,533	40,666,278	42,238,391	40,830,809	42,451,246	41,556,156	40,124,518	37,767,450
Mapping Rate	0.899	0.8973	0.9002	0.9066	0.9067	0.905	0.9085	0.9037	0.9091	0.9144	0.9117	0.907

Note: CC: Chang7–2 under control; CD: Chang7–2 under drought stress; TC: Chang7–2 t under control; TD: Chang7–2 t under drought stress. Raw reads number: the total number of original sequences; clean reads number: the total number of high-quality filtered sequences; clean reads rate (%): the percentage of the number of high quality sequences after filtering to the number of original sequences; raw bases number: the total number of bases in the original off-line sequence; clean bases number: the total number of bases of the filtered high quality sequence; mapped reads: the number of sequences match to the genome; mapping rate: the percentage of sequences match to the genome

Five public databases (NCBInr, NCBInt, UniProtKB, KEGG, and COG) were referred for the annotation of all the detected genes. Approximately 98.78% of detected sequences (31,536) had at least one significant match (*E* < 1e^− 6^) in one of the five databases. One or more gene ontology (GO) terms were assigned to 23,231 genes (72.77%), with 7178 identified GO items belonging to biological process, cellular component and molecular function categories (Table S1). In the biological process category, 7.97, 5.23, and 2.26% of the detected genes were annotated to DNA-templated transcription (GO:0006351), regulation of DNA-templated transcription (GO:0006355) and defense response (GO:0006952), respectively. In the cellular component category, 17.48, 14.88, and 9.75% of genes were annotated as nucleus (GO:0005634), integral component of membrane (GO:0016021), and cytoplasm (GO:0005737), respectively. In the molecular function category, ATP binding (GO:0005524; 11.75%), metal ion binding (GO:0046872; 7.51%) and DNA binding (GO:0003677; 6.11%) ranked highest in GO terms of the detected genes.

**Fig. 3:**
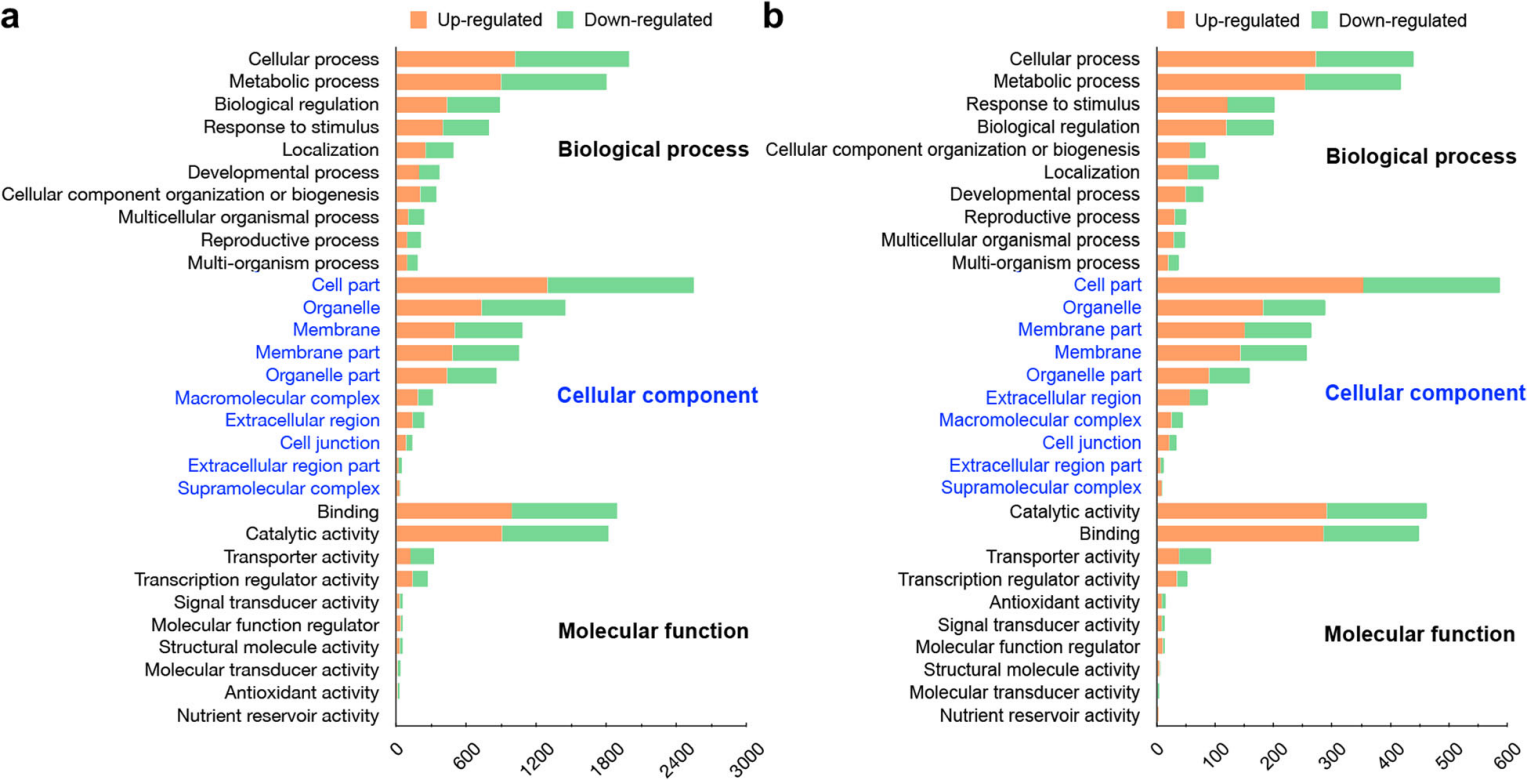
GO functional classification of the DEGs in C7–2 and C7–2t regarding biological processes, cellular components and molecular function. The abscissa stands for the number of genes annotated into GO terms, and the ordinate stands for the GO classification

GO classification of the detected genes in both lines under drought stress displayed a notably high degree of similarity (Fig. 3). In the biological process group, the top three GO categories were cellular process, metabolic process and biological regulation. In the cellular component group, a large number of genes were enriched in cell part, organelle, and membrane. In the molecular function group, the top three GO categories were binding, catalytic activity, and transporter activity.

### Identification of differentially expressed genes (DEGs)

**Fig. 4:**
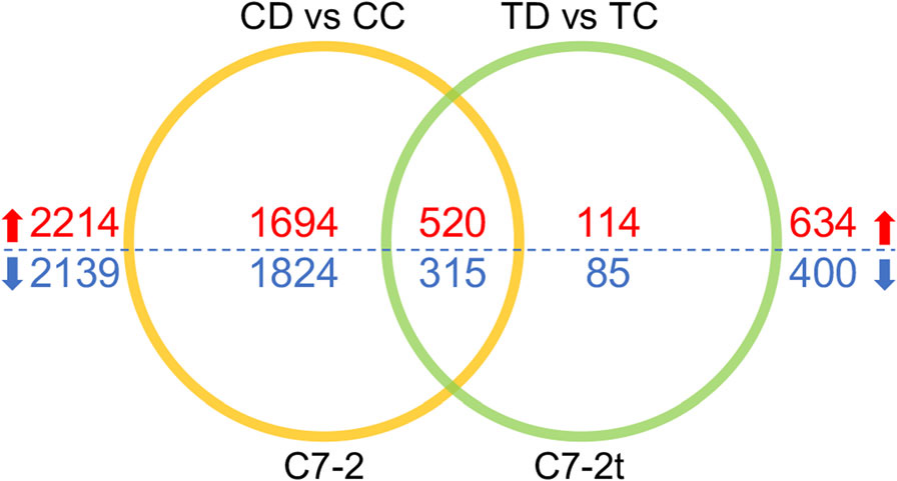
A Venn diagram showing the expression patterns of the DEGs in C7–2 and C7–2t. Red and blue numbers indicate the numbers of the up- and down-regulated genes, respectively

In total, 4353 and 1034 drought-responsive DEGs were identified in C7–2 (CD vs CC) and C7–2t (TD vs TC), respectively (Fig. 4; Table S2). Among the DEGs in C7–2, 2214 were upregulated and 2139 were downregulated. C7–2t, 634 DEGs were upregulated, and 400 were downregulated. A total of 835 genes were shared by the two comparisons (CD vs CC and TD vs TC), including 520 upregulated genes and 315 downregulated genes (Table S3). These DEGs mainly respond to water stress-related stimuli dramatically changed, e.g., the upregulated DEGs *dehydrin*s (*DHN1*, Zm00001d037894; *COR410*, Zm00001d017547, Zm00001d051420) and *universal stress protein A* (Zm00001d034027).

After excluding the shared DEGs, 3518 and 199 DEGs were detected in C7–2 and C7–2t, respectively (Fig. 4; Table S4), suggesting differential drought-responsive pathways between the two lines. Among the 199 DEGs specific to C7–2t, 114 were upregulated and 85 downregulated, which may be related to the drought tolerance of C7–2t. Among the 3518 DEGs specific to C7–2, 1694 were upregulated, and 1824 genes were downregulated. Clearly, more gene expressions were affected by drought stress in C7–2 than in C7–2t. Consequently, the growth of C7–2 plants was significantly inhibited by drought stress compared to C7–2t, suggesting that C7–2t had an active drought adaptation strategy.

### GO and KEGG enrichment of the DEGs

GO enrichment analysis was conducted to investigate the variability in biological processes under drought stress. A total of 182 and 90 GO terms were found as overrepresentations (FDR < 0.05) under drought in C7–2 and C7–2t, respectively (Table S5).

These altered genes in C7–2 primarily affect chloroplast part (GO:0044434) and photosynthesis. The DEGs related to the photosynthetic membrane (GO:0034357) and chloroplast thylakoid membrane (GO:0009535) were significantly enriched. Under drought stress, *Zinc transporter 4* (Zm00001d036965) and *ATP-dependent zinc metalloprotease FTSH 6* (Zm00001d037232) were upregulated; *Photosystem II reaction center PSB28 protein* (Zm00001d000409), *photosynthetic NDH subunit of lumenal location 2* (Zm00001d016943), and *photosynthetic NDH subunit of lumenal location 3* (Zm00001d018623) were downregulated. Moreover, the genes associated with chlorophyll binding (GO:0016168) were significantly suppressed, e.g. *chlorophyll a/b binding protein* (Zm00001d044401; Zm00001d044402; Zm00001d005814; Zm00001d048998) which mainly take part in light harvesting (GO:0009765). Furthermore, the electron transfer processes of photosynthesis were affected (GO:0009773, GO:0009767). Downregulation of these genes mentioned above could explain the decline in photosynthetic efficiency in C7–2 under drought stress (Fig. 2).

With respect to the drought-induced responses in C7–2t, 90 biological processes were enriched under drought (Table S5), much less compared with C7–2, suggesting that C7–2t was relatively less affected by drought stress. It is noteworthy that GO terms related to cell wall were significantly enriched, including plant-type secondary cell wall biogenesis (GO:0009834), plant-type cell wall organization or biogenesis (GO:0071669), cell wall organization or biogenesis (GO:0071554) and plant-type cell wall biogenesis (GO:0009832). Many of the genes involved in these processes were specifically detected in C7–2t, such as *expansin-like A2* (Zm00001d029783), *xyloglucan glycosyltransferase* (Zm00001d038676, Zm00001d020560). In addition, GO terms related to secondary metabolism were also significantly enriched in C7–2t under drought stress, e.g., flavonoid 3′,5′-hydroxylase activity (GO:0033772) and tyrosine ammonia-lyase activity (GO:0052883), which were required in cell wall synthesis.

**Fig. 5:**
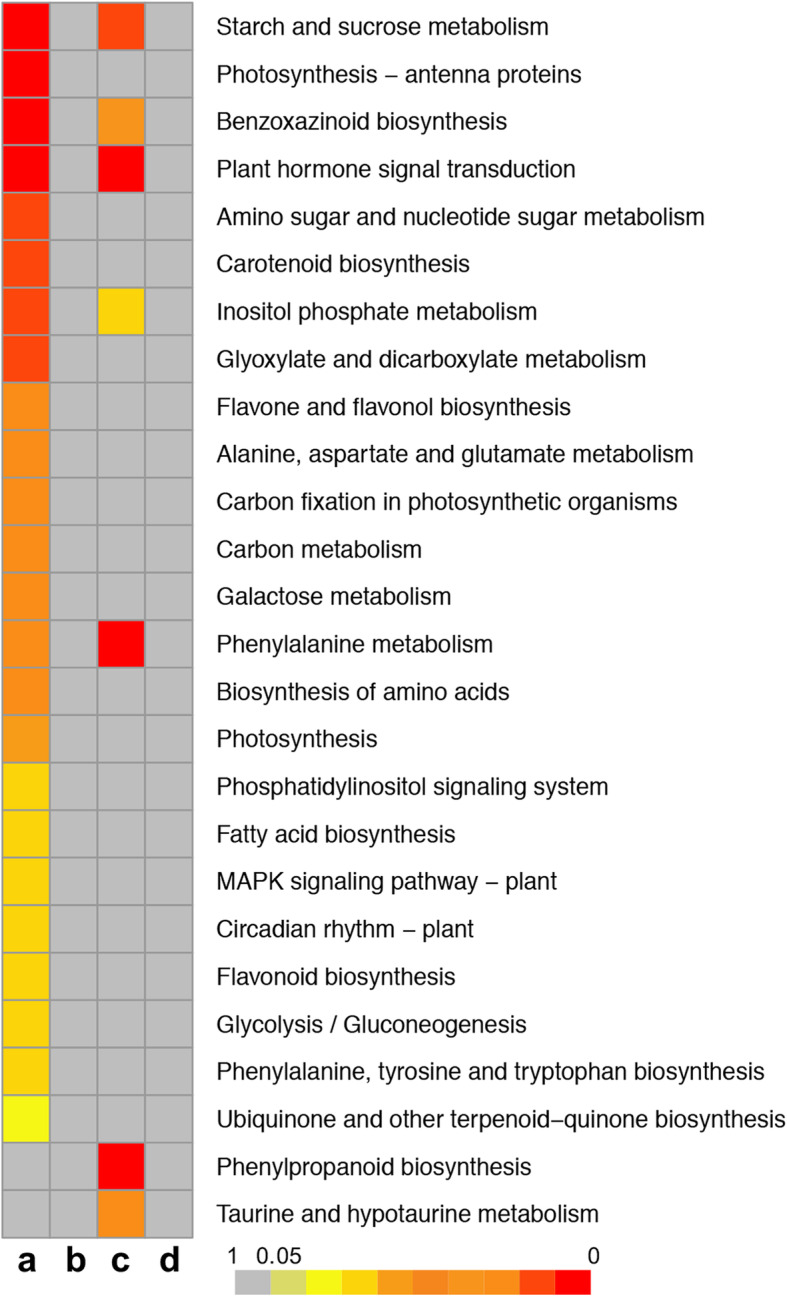
KEGG heatmap of the DEGs in C7–2 and C7–2t. **a** C7–2, drought vs control; **b** C7–2 vs C7–2t under drought stress; **c** C7–2t, drought vs contro0l; **d** C7–2 vs C7–2t under the control conditions. The enrichment degree of each pathway is inversely proportional to *q*-value

A KEGG enrichment analysis was performed to compare the effects of drought on metabolic processes between C7–2t and C7–2 via calculations of the *q-*value. In C7–2t, seven pathways were significantly enriched (Fig. 5; Table S6): plant hormone signal transduction (26 genes), phenylpropanoid biosynthesis (19), phenylalanine metabolism (6), starch and sucrose metabolism (15), taurine and hypotaurine metabolism (4), benzoxazinoid biosynthesis (5), and inositol phosphate metabolism (9). In C7–2, 24 pathways were significantly enriched (Fig. 4; Table S6), of which the top five pathways significantly enriched were starch and sucrose metabolism (50), photosynthesis-antenna proteins (13), benzoxazinoid biosynthesis (14), plant hormone signal transduction (66), and amino sugar and nucleotide sugar metabolism (41). Compared with C7–2, phenylpropanoid biosynthesis and taurine and hypotaurine metabolism were particularly enriched pathways in C7–2t, with eight upregulated and 11 downregulated DEGs involved in the phenylpropanoid biosynthesis pathway and four upregulated genes involved in the taurine and hypotaurine metabolism pathway.

### Cell wall biosynthesis under drought

GO analysis showed that the cell wall (GO:0005618) genes in C7–2t were significantly enriched under drought stress. A total of 23 DEGs in C7–2t were enriched into the entry of plant type cell wall organization or biogenesis (GO:0071669) in the biological process classification, including five downregulated and 18 upregulated. Particularly, the genes encoding expansin proteins participating in cell wall biosynthesis were significantly upregulated, e.g., *expansin-like A2*, *expansin-like A1*, *expansin-B4*, *expansin-B11*, *expansin-B12*, with log2 fold change from 1.1 to 3.29. In addition, the genes (Zm00001d020531; Zm00001d005775; Zm00001d032776; Zm00001d043477; Zm00001d005478; Zm00001d005451) that induced by drought stress encode cellulose synthase A catalytic subunit. These results suggest that C7–2t could reduce the negative effects of drought stress through strengthening the synthesis of cell walls. Although the DEGs encoding expansins were also detected in C7–2, no significant enrichment results were obtained under drought stress.

### Stable sugar synthesis under drought

The DEGs encoding bidirectional sugar transporter (Zm00001d016590; Zm00001d040656; Zm00001d010440; Zm00001d015914; Zm00001d044421) and sucrose synthase (Zm00001d029091; Zm00001d029087; Zm00001d045042) were all up-regulated in C7–2 and C7–2t. The upregulation of sugar synthesis and transport under drought requires a steady supply of energy from photosynthesis.

In C7–2, drought-responsive 69 genes were involved in photosynthesis (GO:0015979), photosynthesis and dark reaction (GO:0019685), photosynthesis, light harvesting, photosynthesis (GO:0009765), light harvesting in photosystem I (GO:0009768), and other biological process. Among them, 66 DEGs were inhibited by drought, but only three were induced by drought (Zm00001d042211, Zm00001d001820, *protochlorophyllide reductase A*; Zm00001d016166, *phosphoenolpyruvate carboxylase*).

In C7–2, the expressions of the DEGs encoding triose phosphate/phosphate translocator under drought were inconsistent: one up-regulated (Zm00001d005542), two down-regulated (Zm00001d032383; Zm00001d039258). A gene encoding sucrose synthase (Zm00001d051837) and several genes encoding sugar transporter were down-regulated (Zm00001d029251, Zm00001d009603, Zm00001d029254, Zm00001d009605). The downregulation expression of these genes was not detected in C7–2t. These results suggested that the stable photosynthesis of C7–2t under drought stress may provide a better guarantee for the synthesis and metabolism of sugars.

### qRT-PCR verification of the DEGs

**Fig. 6:**
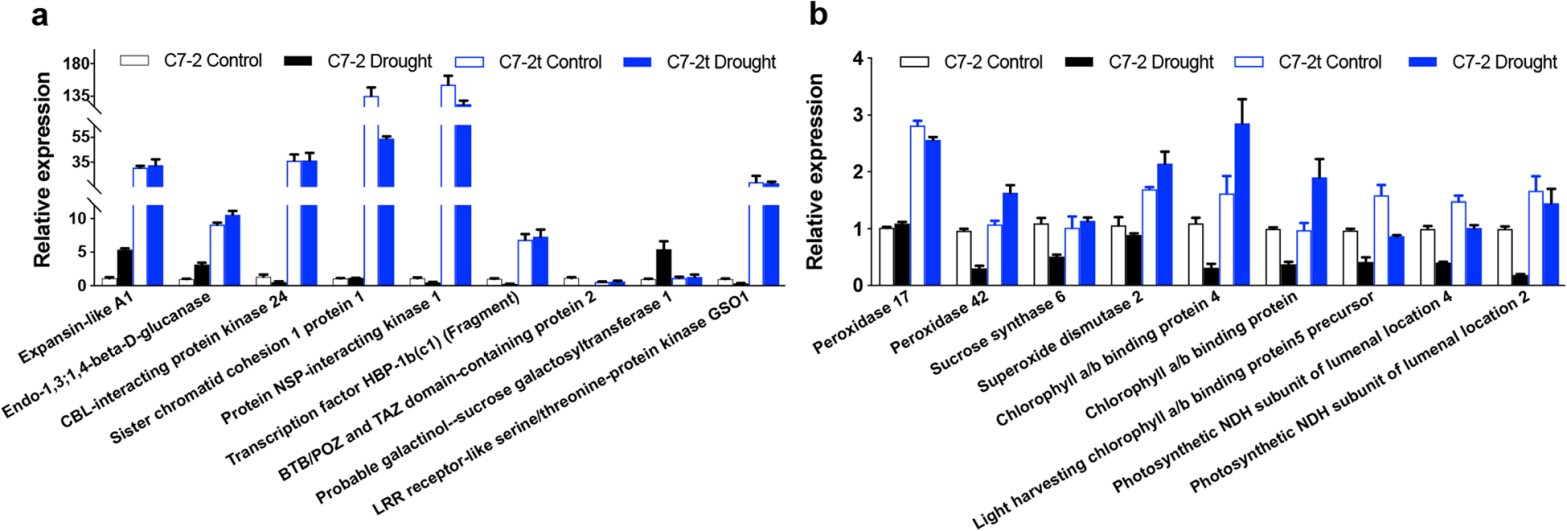
qRT-PCR comparison of the expression of 18 drought-responsive DEGs between C7–2 and C7–2t

A total of 18 DEGs of interest were selected to evaluate the accuracy of the RNA-seq results (Table S7) with qRT-PCR (Fig. 6). In particular, the expression level of *sucrose synthase 6* was decreased in C7–2 under drought; the expression levels of *peroxidase 17*, *peroxidase 42*, *superoxide dismutase 2* and other five DEGs involved in photosynthesis were higher in C7–2t than those in C7–2. The results of qRT-PCR were consistent with those of the transcriptomic analysis.

## Discussion

### Differentially physiological and biochemical responses between C7–2t and C7–2

In the present study, a drought-tolerant maize mutant C7–2t by ^60^Co-γ irradiation was developed. Compared to its wild type C7–2, C7–2t exhibited a higher drought tolerance under drought stress. There were significant differences in physiological and biochemical indexes between C7–2t and C7–2 (Fig. 2).

RWC is widely used to identify the drought tolerance of various plant species, including maize [20, 38]. Plants with higher RWC can increase their adaptability by reducing the drought-caused damage to their antioxidant systems [39]. C7–2t showed a higher RWC in leaves, i.e. a high moisture retention ability, than C7–2 under drought. Proline and soluble sugar plays important roles in osmotic regulation [40], correlating with drought tolerance [41, 42]. Under drought, C7–2t accumulated more proline and soluble sugars in the leaves than those in C7–2.

An improved efficiency of the antioxidative defense system could protect photosynthetic pigments, proteins, and DNA from excess ROS damage [40, 43]. CAT activity is inversely proportional to MDA content under severe drought stress [20]. The assay of CAT activity and MDA content indicated that C7–2t suffered less ROS damage than C7–2 under drought stress.

In the field, light distribution in plant canopy can be improved by reducing Chl content [44, 45]. On the other hand, nitrogen and energy saved by reducing Chl synthesis would enhance the specific responses to drought stress [46]. Thus, low Chl content in the leaves of C7–2t might be a part of its drought adaptation strategy. Taken together, higher levels in RWC, osmolyte accumulation, antioxidant activities and photosynthetic efficiency and a less level of MDA contributed to higher drought tolerance of C7–2t than C7–2.

### The contribution of osmoregulation of soluble sugars to drought tolerance of C7–2t

Soluble sugars, including glucose, sucrose, maltose, and trehalose, play active roles in osmoregulation under drought stress [47–49]. In a drought-resistant wheat, the increased expression of soluble sugar synthesis-related genes explained its drought tolerance [50]. In the present study, the soluble sugar content in C7–2t was higher than that in C7–2 under the control and drought stress conditions (Fig. 2). Clearly, C7–2t has an efficient drought adaptation strategy partly based on osmoregulation of soluble sugars. In the present study, the expression of photosynthesis-related genes in C7–2t was almost unaffected, thus C7–2t could synthesize soluble sugar more efficiently under drought conditions.

C7–2t regulated the specific component of soluble sugar at the transcriptome level. Glucose could form trehalose with a 1,1-glycosidic linkage and reduce the injury caused by high concentrations of trehalose-6-phosphate under drought stress [48]. In the present study, the expression of *trehalose 6-phosphate synthase/phosphatase and trehalose 6-phosphate phosphatase* showed a sharp fluctuation in C7–2 (log_2_ fold changes from − 3.46 to 7.63) under drought stress, whereas their expressions were inhibited in C7–2t, implying that the elevated content of soluble sugars in C7–2t might not be due to the enhanced trehalose synthesis.

Moreover, the expression of *β-amylase* (Zm00001d014083) was activated under drought in both C7–2 and C7–2t, indicating that starch in the leaves could be decomposed into maltose. However, several genes encoding β-amylase in C7–2 were downregulated under drought (Zm00001d009016, Zm00001d029983, Zm00001d047077), possibly resulting in an decreased content of maltose and an increased sensitivity of photosynthetic system to drought stress [51]. In summary, C7–2t could synthesize more soluble sugars in its leaves and maintain the stability of photosynthesis under drought stress, thereby making it more drought-tolerant than C7–2.

### The contribution of cell wall biosynthesis-related genes to water retention in C7–2t

The involvement of an organized multienzyme complex in polysaccharide biosynthesis provided a strong guarantee for cell wall synthesis [52]. The elevated expression of *glycosyltransferase* contributed to drought resistance of *Arabidopsis thaliana* cytokinin-deficient mutants [53]. In the present study, several *glucuronosyltransferases* in C7–2t (Zm00001d039231, Zm00001d007231) and C7–2 (Zm00001d002064, Zm00001d007231, Zm00001d008250) were upregulated under drought stress. GO enrichment analysis revealed that in C7–2t many DEGs were related to cell wall organization, especially the *expansin* family [54] that was induced by various abiotic stresses and ABA [55, 56]. Stomatal density was shown to decrease by overexpression of *RhEXPA4* [57]. Furthermore, expansins could reduce water loss by discontinuing cell wall activity and stiffening cell structures [27].

The lignin content and intermediate product of the phenylpropanoid synthesis pathway were positively correlated with drought tolerance in maize [58, 59]. The accumulation of caffeic acid and *p*-coumaric acid in the xylem sap regulated the growth of maize leaves by influencing lignin biosynthesis under drought stress [60, 61]. *p*-Coumaric acid could rapidly lignify tissues under stress and improve adaptability under drought stress [62, 63]. In the present study, KEGG and GO enrichment analyses showed that lignin-related metabolic activities, such as phenylpropanoid synthesis pathway, were enhanced in C7–2t under drought. Most possibly, phenylpropanoid biosynthesis pathway and its intermediates, caffeic acid and *p*-coumaric acid, were involved in the response of C7–2t to drought stress.

### The role of ABA in drought adapation strategy in C7–2t

9-cis Epoxycarotenoid dioxygenase 1 was a key enzyme involved in ABA synthesis [64, 65]. In the present study, the log_2_ fold change of the genes encoding 9-cis epoxycarotenoid dioxygenase 1 (Zm00001d018819, Zm00001d033222) reached 4.48 and 3.15 in C7–2t, whereas the corresponding changes were 7.03 and 4.57 in C7–2, suggesting that the sensitivity of C7–2 to drought stress may result from more endogenous ABA produced under drought stress.

PP2C participates in the ABA signal transduction pathway as a negative regulator under stress [26]. A recent study proved that *PP2C-A10* (localized on chromosome 6) was related to ER stress [26]. In the present study, 29 PP2C genes (25 upregulated and 4 downregulated) and 14 (all upregulated) were detected in C7–2 and C7–2t, respectively. Especially, the log_2_ fold change of *PP2C-A10* was significantly lower in C7–2t than in C7–2. These results that ABA-dependent upregulated genes provided transcriptional support for drought tolerance in plants [6], including C7–2t.

## Conclusions

**Fig. 7:**
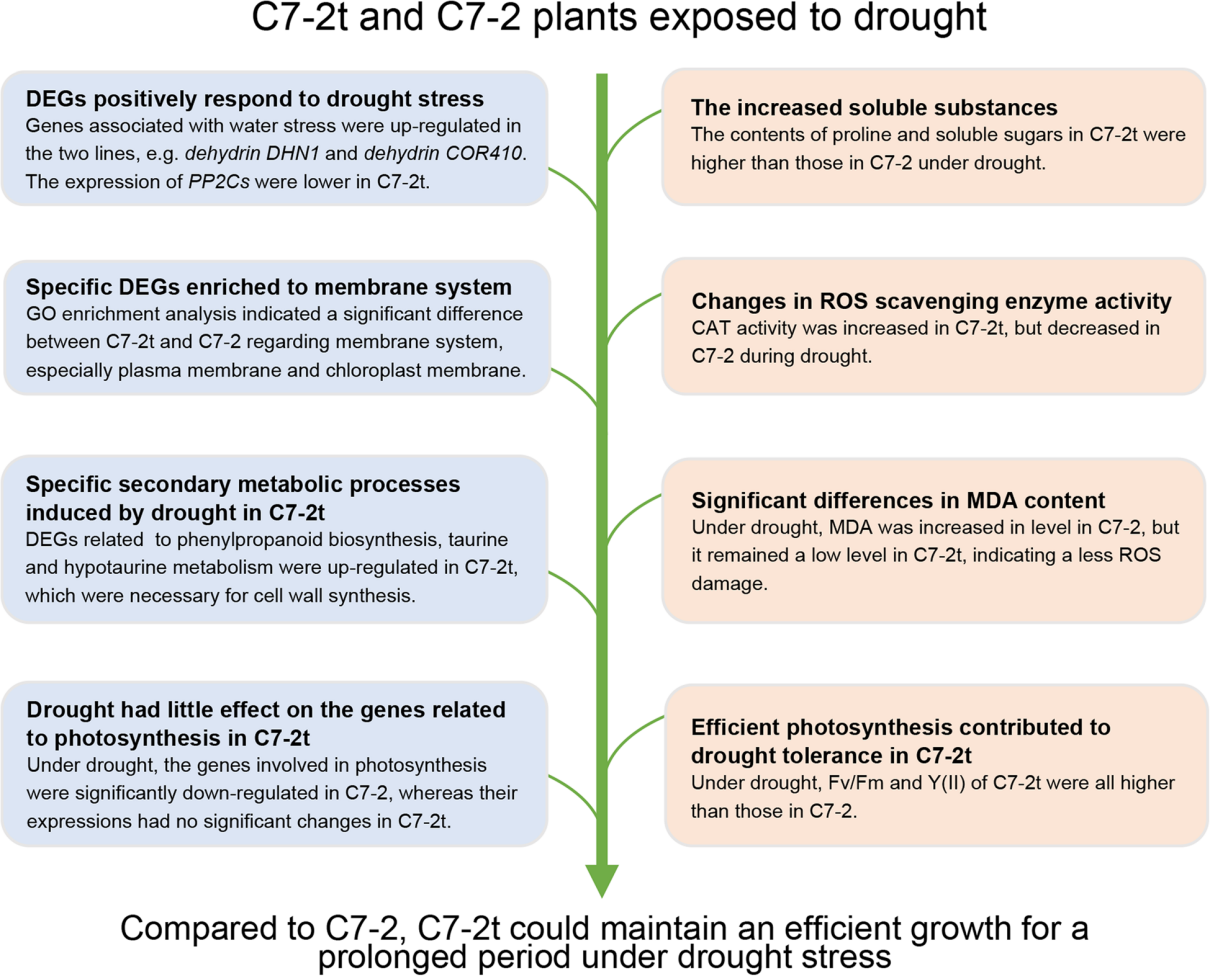
Possible drought-adaptation mechanisms in the drought-tolerant mutant C7–2t

In the present study, we developed a more drought-tolerance inbred line C7–2t compared with C7–2 in both controlled and field conditions. The differences in physiological, biochemical and transcriptomic changes between C7–2 and C7–2t could explain the drought tolerance mechanisms in C7–2t (Fig. 7). The drought-tolerant mutant C7–2t and the drought-responsive DEGs identified here will be useful for basic research and drought tolerance breeding in maize.

## Methods

### Creation of drought-tolerant maize mutants

The seeds of maize (*Zea mays* L.) C7–2 were purchased from Henan Qiule Seed Industry Science and Technology Company, Ltd. (Zhengzhou, China). The seeds were artificially mutated by ^60^Co-γ irradiation for 1 h with three doses of 150, 200 and 250 Gy. The 200 Gy radiation had a fatality rate of approximately 50%. Thus, the 200 Gy-treated seeds were planted in the field in 2013 to produce the M_0_ generation. Through reducing watering times to maintain soil drought, the drought-tolerant plants were selected and self-pollinated. Field selection process was continuously performed for five years (mainly by QBZ and WW). Since M_4_ generation, the promising drought-tolerant lines (such as C7–2t) that performed better than C7–2 in the field were collected and preserved in seed storage room (College of Life Sciences, Henan Agricultural University, China). The drought tolerance of C7–2t was further evaluated in pot experiments, based on phenotypes, physiological and biochemical changes during drought [66].

### Maize materials and growth conditions

The seeds were washed with 2% sodium hypochlorite for 10 min and rinsed with distilled water three times. The samples were then planted in a plastic box filled with 1100 g of the growing medium (Pindstrup Substrate, 0–6 mm, pH 6.0, Pindstrup Mosebrug A/S, Denmark) and 1000 ml of water. The boxes were placed into a growth chamber with 27 °C, 60% humidity and 14 h of light and 10 h of darkness. Seven-day-old seedlings were divided into two groups: (a) control plants with normal watering and (b) drought-treated plants that were subjected to stopping watering for seven days. Afterwards, the expanded third leaves were collected for physiological, biochemical and RNA-seq analysis. Leaves from three individual plants represented a sample and were stored at − 80 °C after being frozen in liquid N_2_. All experiments were performed in at least three independent biological replicates.

### Physiological and biochemical assays

Fresh leaves were divided into two equal groups: one was dried at 80 °C for 12 h to determine dry weight, and the other was placed in distilled water in the dark for 12 h to determine the turgid weight. RWC was calculated by the following equation: RWC = (fresh weight - dry weight) / (turgid weight - dry weight) · 100% [67]. CAT activity and Chl, free proline, SSC and MDA content were assayed by the established methods [68–72]. Chl fluorescence was determined by a MINI-PAM-II (Walz, Germany). The maximum photochemical quantum yield (F_v_/F_m_) and effective photochemical quantum yield (Y(II)) of PS II were calculated [73, 74]. All assays were performed in three biological experiments.

### RNA extraction and detection

Twelve samples in three biological replicates were used to extract RNA using an Ultrapure RNA Kit (CoWin Biotech Co., China) according to the manufacturer’s instructions. The RNA was treated with RNase-free DNase I (Takara, Japan) to remove any possible DNA. The integrity was then checked by gel electrophoresis and an Agilent 2100 Bioanalyzer (Agilent Technologies, Palo Alto, CA, USA). The concentrations of total RNA were determined using a NanoDrop 8000 spectrophotometer (NanoDrop, Wilmington, DE). Total RNA with a RIN value ≥7.3 and a 28S:18S ratio ≥ 1.2 was subjected to RNA-seq analysis by Annoroad Gene Technology Co., Ltd. (Beijing, China).

### RNA-seq analysis

About 2 μg of RNA per sample was used as input material for RNA-seq analysis. Sequencing libraries were generated using a NEBNext® Ultra™ RNA Library Prep Kit for Illumina® (#E7530L, NEB, USA) following the manufacturer’s recommendations. Index codes were added to attribute the sequences in each sample. Briefly, mRNA was purified from the total RNA using poly-T oligo-attached magnetic beads. Fragmentation was carried out by divalent cations under elevated temperature in NEBNext First Strand Synthesis Reaction Buffer (5X). RNase H and polymerase I were used for catalytic first-strand cDNA and second-strand cDNA syntheses, respectively. A-tailing and adapters were implemented after purification and terminal reparation. The purified cDNA template was enriched by PCR, and then the library was completed. Cleaned RNA-seq reads were obtained from the raw reads after removing the contaminated reads, low-quality reads and reads whose N base was greater than 5% for the total bases.

The reference genomes and the annotation file were downloaded from the ENSEMBL database (http://www.ensembl.org/index.html). Bowtie2 was used to construct the genome index. The clean data were aligned to the reference genome by HISAT2 [75, 76], and gene expression was calculated by FPKM [77]. DEGs were estimated by the software DESeq2, after which *p*-values were calculated according to the Wald test [78]. The *p*-values were corrected by the BH method. Genes with *q* ≤ 0.05 and |log2_ratio| ≥ 1 were identified as DEGs.

### Functional enrichment analysis

GO terms with FDR < 0.05 were considered to be significantly enriched. Kyoto Encyclopedia of Genes and Genomes (KEGG, http://www.kegg.jp/) enrichment of the DEGs was implemented by hypergeometric test, in which *p*-values were adjusted by multiple comparisons as *q*-values. KEGG terms with *q* < 0.05 were considered to be significantly enriched.

### Quantitative qRT-PCR analysis

Twelve total RNA samples were extracted from three independent groups for each line under drought and control conditions. First-strand cDNA synthesis were performed with SuperReal PreMix Plus (Tiangen, Beijing, China).

A total of 18 genes were selected, and gene-specific primers were designed with the online tool Primer3 (http://primer3.ut.ee). The ubiquitin gene was chosen as a loading control in conjunction with primers Ubi 5′ (5′-TAAGCTGCCGATGTGCCTGCG-3′) and Ubi 3′ (5′-CTGAAAGACAGAACATAATGAGCACAG-3′). Each PCR (20 μL) contained 10 μL of 2× SuperReal PreMix Plus, primers at 0.6 μM and appropriately diluted cDNA. qRT-PCR was performed using Thermo Fisher Scientific StepOnePlus™ Real-Time PCR Instrument according to the manufacturer’s instructions. The thermal cycling conditions were 95 °C for 15 min followed by 40 cycles of 10 s at 95 °C and 32 s at 60 °C. At the second dissociation stage, 95 °C for 10 s followed by 65–95 °C with increments of 0.5 °C for 0.05 s were used. All reactions were performed in triplicates. The relative expression levels were calculated by the 2^−ΔΔCT^ method [79].

## Supplementary information

**Additional file 1 **: **Table S1** FPKM value and annotation of all the genes detected from RNA sequencing.

**Additional file 2 **: **Table S2** The DEGs detected in C7–2 and C7–2t.

**Additional file 3 **: **Table S3** The common DEGs shared by C7–2 and C7–2t.

**Additional file 4 **: **Table S4** The DEGs specific to C7–2 and C7–2t.

**Additional file 5 **: **Table S5** GO enrichment analysis of the DEGs in C7–2 and C7–2t.

**Additional file 6 **: **Table S6** KEGG enrichment analysis of the DEGs in C7–2 and C7–2t.

**Additional file 7 **: **Table S7** Primers used for qRT-PCR.

**Additional file 8 **: **Fig. S1** Comparison of drought tolerance index, barren ear tip distance and ASI between C7–2 and C7–2t. (**a**) Evaluation via pot experiments in third-leaf stage of seedlings. (**b**) and (**c**) Showing the difference in ear traits and the abscissa standing for ASI.

**Additional file 9 **: **Fig. S2** Plant height, ear height and biomass of C7–2 and C7–2t at harvest in the field.

## Data Availability

All data generated or analyzed during this study are included in this published article and its supplementary information files. The raw datasets of transcriptomic analysis are available from the corresponding author on reasonable request after the publication of the work.

## Abbreviations

ASI: Anthesis-silk interval
C7–2: Inbred line Chang7–2
C7–2t: A drought-tolerant inbred line
CAT: Catalase
CC: C7–2 control
CD: C7–2 drought treatment
Chl: Chlorophyll
DEGs: Differentially expressed genes
FPKM: Fragments per kilobase of exon model per million reads mapped
GO: Gene ontology
MDA: Malondialdehyde
ROS: Reactive oxygen species
RWC: Relative water content
SSC: Soluble sugar content
TC: C7–2t control
TD: C7–2t drought treatment
