# Supplementary material for: Identification of drought tolerant mechanisms in a drought-tolerant maize mutant based on physiological, biochemical and transcriptomic analyses
Source: BMC Plant Biol. 2020 Jul 9;20:315. doi: 10.1186/s12870-020-02526-w (PMC7350183; doi:10.1186/s12870-020-02526-w)
Supplement: Supplementary file 7 — Additional file 7 : Table S7 Primers used for qRT-PCR. [file 12870_2020_2526_MOESM7_ESM.docx]

Table S7 Primers used for qRT-PCR

| **Gene** | **Description** | **F****orward Primer** | **Reverse Primer** | **Target Size bp** |
| --- | --- | --- | --- | --- |
| Zm00001d048344 | Expansin-like A1 | CTGAAGAGCCTGCCTGTAGAAT | TACTTATCCGACACGTCAGCAG | 202 |
| Zm00001d010343 | Endo-1,3;1,4-beta-D-glucanase | TACTTGGAGCTGAGATGGATGTG | ATCATCGAGGCTGTATCTCACG | 149 |
| Zm00001d000407 | CBL-interacting protein kinase 24 | GAAGTAACGCACAAGGATGGTG | CCATTGACTCAGCAACAACCTC | 190 |
| Zm00001d039132 | Sister chromatid cohesion 1 protein 1 | CTGCCGATGGATTTAACAGATGG | CCACTTCCTGATGGCAATTGTAC | 206 |
| Zm00001d045729 | Protein NSP- interacting kinase 1 | TGTACCTGGTCCTTCATCTCCT | CTTTGCTAACTTGCCGATCTCTG | 183 |
| Zm00001d043153 | Transcription factor HBP-1b(c1) (Fargment) | TAGATCAGAAGACACTTCGGCG | AATACCCTGTTGACGAGTCTGG | 155 |
| Zm00001d025707 | BTB/POZ and TAZ domain-containing protein 2 | GAAACTGAGATACTCCCGGTCC | GTTTCAGACAGACCTCATGACCT | 125 |
| Zm00001d031300 | Probable galactinol--sucrose galactosyltransferase 1 | ACATTCTGAACTGGTTTGGCTG | TTGTGGTTCTCCCTGATGTGAG | 232 |
| Zm00001d007750 | LRR receptor-like serine/threonine-protein kinase GSO1 | ATCAGGTAGCATCCCGACAATC | CTGGGATTGGACCGTAGAACAT | 164 |
| Zm00001d020808 | Peroxidase 17 | TCGAGGGGATGATAAAGATGGG | TCGAGGGGATGATAAAGATGGG | 179 |
| Zm00001d022456 | Peroxidase 42 | GTACAGCAAGAAGAACCTCGAC | GTACAGCAAGAAGAACCTCGAC | 237 |
| Zm00001d051837 | Sucrose synthase 6 | CAAGGCTCATACCAGAAGCAAAG | GTACAAGAATCCTGGGCGTATCT | 184 |
| Zm00001d036135 | Superoxide dismutase 2 | CAGAGTATAACAACGCAGCACAG | AACCAGACCCTAACAGCTGTAAG | 180 |
| Zm00001d032197 | Chlorophyll a/b binding protein 4 | CTGGAAGGCAAACATTGAAACC | ACGATCTCTACCCTTAATATCCCC | 180 |
| Zm00001d033132 | Chlorophyll a/b binding protein | GTTCTCCATGTTCGGCTTCTTC | AGAGAAGACCACACTGTTCACTC | 223 |
| Zm00001d007267 | Light harvesting chlorophyll a/b binding protein5 precursor | GAAACACCCTCAGCTATTTCGG | CGTTCTTGATCTCCTTCACCTTG | 216 |
| Zm00001d018077 | Photosynthetic NDH subunit of lumenal location 4 | TATATGACCTTTTCCTCGTCGGG | CTTTCACCGCCAAGAATTCCTC | 102 |
| Zm00001d016943 | Photosynthetic NDH subunit of lumenal location 2 | GAAAACGTTGAAGTCGCTGGAT | TTTCATTCTTAGACGCACGGAG | 113 |
