# Supplementary figures and images for: Identification of drought tolerant mechanisms in a drought-tolerant maize mutant based on physiological, biochemical and transcriptomic analyses
Source: BMC Plant Biol. 2020 Jul 9;20:315. doi: 10.1186/s12870-020-02526-w (PMC7350183; doi:10.1186/s12870-020-02526-w)

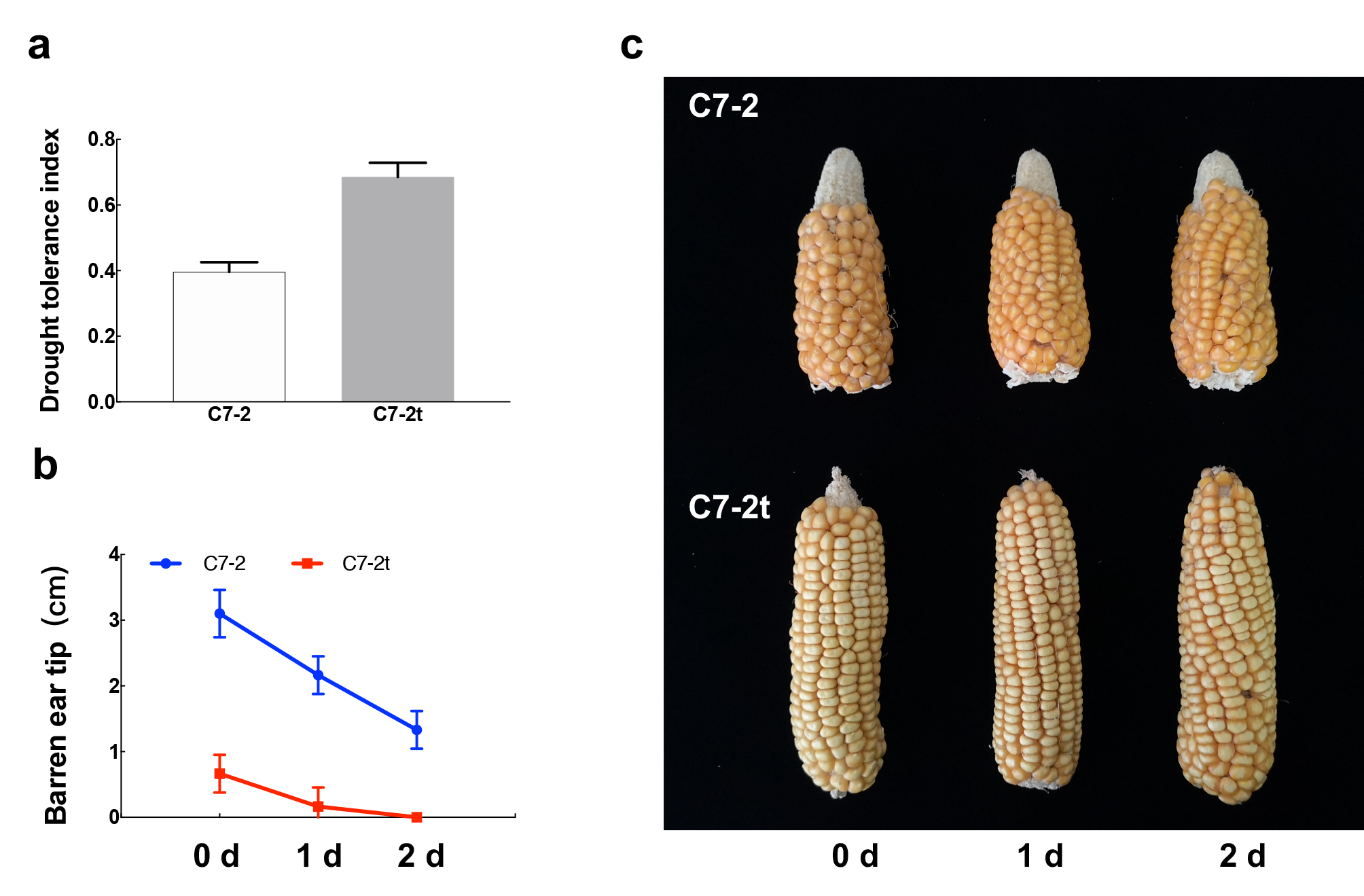

Supplement: Supplementary file 8 — Additional file 8 : Fig. S1 Comparison of drought tolerance index, barren ear tip distance and ASI between C7–2 and C7–2t. (a) Evaluation via pot experiments in third-leaf stage of seedlings. (b) and (c) Showing the difference in ear traits and the abscissa standing for ASI. [file 12870_2020_2526_MOESM8_ESM.tif]

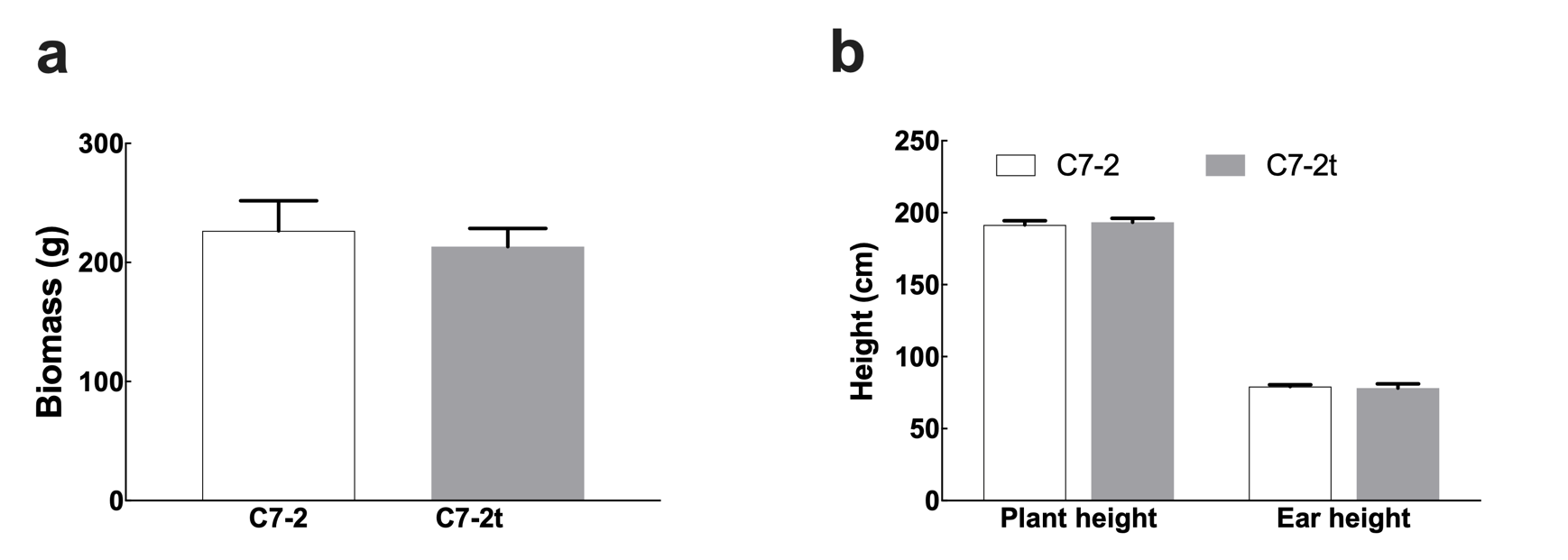

Supplement: Supplementary file 9 — Additional file 9 : Fig. S2 Plant height, ear height and biomass of C7–2 and C7–2t at harvest in the field. [file 12870_2020_2526_MOESM9_ESM.tif]
